# Data on scaling up and in vivo human study of progesterone lipid nanoparticles

**DOI:** 10.1016/j.dib.2017.08.033

**Published:** 2017-08-31

**Authors:** Elisabetta Esposito, Maddalena Sguizzato, Markus Drechsler, Paolo Mariani, Federica Carducci, Claudio Nastruzzi, Rita Cortesi

**Affiliations:** a Department of Life Sciences and Biotechnology, University of Ferrara, I-44121 Ferrara, Italy; b BIMF/Soft Matter Electronmicroscopy, University of Bayreuth, Germany; c Department of Life and Environmental Sciences and CNISM, Università Politecnica delle Marche, I-60100 Ancona, Italy

## Abstract

Progesterone containing nanoparticles constituted of tristearin or tristearin in association with caprylic-capric triglyceride were produced in a lab scale by ultrasound homogenization and in a pilot scale by high pressure homogenization. A study was conducted to select the pressure to be used in order to obtain homogenously sized nanoparticles. The Dialysis method was performed to mimic subcutaneous administration of lipid nanoparticles. Mathematical analyses of the results were conducted to understand and compare the drug release mechanisms.

A human in vivo study, based on tape stripping, was conducted to investigate the performance of nanoparticles as progesterone skin delivery systems. Tape stripped *stratum corneum* was analyzed by light microscopy.

**Specifications Table**

**Table:** 

Subject area	*pharmaceutical chemistry; dermatology; nanotechnology*
More specific subject area	*Drug delivery; nanoparticles; cutaneous administration*
Type of data	*Table, image, graph*
How data was acquired	*HPLC using an Agilent Zorbax Eclipse XBD-C18 column (Agilent Technologies, United States) (15 cm×0.46 cm) stainless steel packed with 5* *um particles. Light Microscope, Nikon E800*
Data format	*Raw, analyzed*
Experimental factors	*ultrasound homogenization and high pressure homogenization process; Stratum corneum portions excised by tape stripping*
Experimental features	*Lipid nanoparticles were alternatively prepared by hot homogenization techniques based on ultrasound or high pressure. Release experiments were performed by dialysis method and HPLC. Stratum corneum was imaged by optical microscopy at 40× magnification*
Data source location	*Ferrara, Italy, Via Fossato di Mortara 19*
Data accessibility	*data are available with this article*
Related research article	*Progesterone lipid nanoparticles: scaling up and in vivo human study*

**Value of the data**

• The images explain how lipid nanoparticles have been prepared.
• The table summarized the effect of pressure on size of nanoparticle produced by high pressure homogenization, it can help the reader to choose the pressure.
• The graph indicates the mechanism of progesterone release from nanoparticles.
• *Stratum corneum* images show the differences between the different tapes stripped from the skin.

## 1 Data

Fig. S1 shows the apparatuses employed for nanoparticle production. Particularly panels A and C refer to the first step based on emulsification of lipid phase in water phase by ultraturrax, while panels B and D refer to the second step, respectively to ultrasound treatment and to high pressure homogenization.

Lipid nanoparticles containing progesterone have been gelled by xanthan-gum addition and applied on the skin to quantify the presence of progesterone on the *stratum corneum*. Fig. S2 reports the images of *stratum corneum* sections stripped from forearms of volunteers by tapes. After seven sequential stripping on the same site the presence of *stratum corneum* cells is detectable.

For nanoparticle production by high pressure homogenization, an initial emulsion was obtained by dispersing the fused lipids in aqueous solution under high speed stirring at 15000 rpm for 1–3 min. Table S1 reports particle mean diameters, measured by PCS after cooling the emulsion at 25 °C. Varying the emulsification time (in the 1–3 min range) caused only marginal variations in mean diameter of the obtained particles.

The influence of pressure in the production of nanoparticles by high pressure homogenization was considered. The range 40–120 MPa was investigated (Fig. S3); pressure level from 40 to 100 MPa decreased the size, higher pressure levels, up to 120 MPa increased the particle dimension.

A mathematical analysis of the release profile was conducted to determine the mechanism of progesterone release form nanoparticles. Theoretical release profiles were calculated accordingly to mimicking dissolutive and diffusive model. Fig. S4A compares the theoretical and experimental release from progesterone containing solid lipid nanoparticles while Fig. S4B refers to progesterone containing nanostructured lipid carriers.

In Fig. S4A the experimental curve is almost superimposable to the diffusive theoretical curve; suggesting a release with a fickian diffusive mechanism [1], [2]. In Fig. S4B the experimental curve is indeed partly overlapping the theoretical dissolutive curve and partly the theoretical diffusive curve, indicating a mixed release mechanism [3].

## 2 Experimental design, materials, and methods

The copolymer poly (ethylene oxide) (a) –poly (propylene oxide) (b) (*a*=80, *b*=27) (poloxamer 188) was a gift of BASF ChemTrade GmbH (Burgbernheim, Germany). Miglyol 812N, caprylic/capric triglycerides (miglyol) was a gift of Cremer Oleo Division (Witten, Germany). Tristearin, stearic triglyceride (tristearin), progesterone (PRG) and HPLC solvents were purchased from Sigma-Aldrich, Merck (Darmstadt, Germany).

Lipid nanoparticles were alternatively prepared by hot homogenization techniques based on ultrasound or high pressure. In both cases the lipid phase (5% with respect to the whole weight of the dispersion) was constituted of pure tristearin (in the case of solid lipid nanoparticles, SLN preparation) or a mixture of tristearin and miglyol in a 2:1, w/w ratio (in the case of nanostructured lipid carriers, NLC preparation), while the aqueous phase was a poloxamer 188 solution (2.5%, w/w).

### 2.1 Ultrasound homogenization (UH) method

As first step an emulsion was obtained by addition of the aqueous phase (4.75 ml) heated at 80 °C to the molten lipid phase (250 mg) followed by mixing at 15,000 rpm, 80 °C for 1, 2 or 3 min (IKA T25 digital ultra-turrax) (Fig S1A). As second step, the emulsion was subjected to ultrasound homogenization at 6.75 kHz for 5, 10 or 15 min (Microson ultrasonic Cell Disruptor-XL Minisonix) (Fig S1B) and let cooling at 25 °C. Lipid nanoparticle dispersions were stored at room temperature.

### 2.2 High pressure homogenization (HPH) method

As first step, the aqueous phase (950 ml) heated at 80 °C was added to the molten lipid phase (50 g) and subjected to stirring at 15,000 rpm, 80 °C for 1, 2 or 3 min (Fig S1C). The emulsion was homogenized using high pressure homogenizer for 1–3 cycles at 100 MPa (Panda Plus2000/GEA Niro Soavi, Parma, Italy) to form o/w nanoemulsion (Fig S1D). The nanoemulsion was then cooled by a coil employed as heat exchanger directly connected to the instrument for solidification and formation of lipid nanoparticles. Lipid nanoparticle dispersions were stored at room temperature.

Both for UH and HPH methods, in the case of PRG loaded nanoparticles (named SLN-PRG and NLC-PRG), the drug (0.1% w/w with respect to the whole dispersion, 0.02% w/w with respect to the lipid phase) was added to the fused lipid phase before emulsification step.

### 2.3 Photon Correlation Spectroscopy (PCS) analysis

Submicron particle size analysis was performed using a Zetasizer 3000 PCS (Malvern Instr., Malvern, England) equipped with a 5-mW helium neon laser with a wavelength output of 633 nm. Glassware was cleaned of dust by washing with detergent and rinsing twice with water for injections. Measurements were made at 25 °C at an angle of 90° periodically from 0 to 6 months after nanoparticle production. Data were interpreted using the “CONTIN” method [4].

### 2.4 in vitro dialysis release studies

in vitro dialysis release studies were performed on PRG alternatively solubilized in SLN-PRG, NLC-PRG or suspended in poloxamer 188 solution (2.5%, w/w). Two milliliters of solution/suspension (PRG 0.1%, w/w) were put into a dialysis tube (6 cm) (molecular weight cut off 10,000–12,000; Medi Cell International, England), then placed into 30 ml of receiving phase constituted of phosphate buffer (100 mM, pH 7.4) and ethanol (70:30, v/v) and shaken in a horizontal shaker (MS1, Minishaker, IKA) at 175 rpm at 37 °C. Samples of receiving phase were withdrawn at regular time intervals, and analyzed by a HPLC method using an Agilent Zorbax Eclipse XBD-C18 column (Agilent Technologies, United States) (15 cm×0.46 cm) stainless steel packed with 5 um particles, eluted at room temperature with different mobile phases. Samples of 50 ul were injected through the rheodyne injector system fitted with 50 ul fixed loop and compared with standards of known concentration. The mobile phase composition was methanol/water (80:20, v/v), with flow rate 1 ml/min at 220 nm. Fresh receiving mixture was added to maintain constant volume. The PRG concentrations were determined six times in independent experiments and the mean values±standard deviations were calculated.

The experimental data obtained by the release experiments were fitted to the following semi-empirical equations respectively describing Fickian dissolutive (1) and diffusion (2) release mechanisms [3], [5]

(1) $$Mt/M\infty =K_{Diss}t^{0.5}+c$$

(2) $$1-Mt/M\infty =e^{-Kdiff}t+c$$

where Mt/*M*∞ is the drug fraction released at the time *t*, (*M*∞ is the total drug content of the analyzed amount of SLN), *K* and *c* are coefficients calculated by plotting the linear forms of the indicated equations. The release data of percentage of released drug (0–8 h) were used to produce theoretical release curves.
