# Supplementary figures and images for: Data on scaling up and in vivo human study of progesterone lipid nanoparticles
Source: Data Brief. 2017 Aug 31;14:639–42. doi: 10.1016/j.dib.2017.08.033 (PMC5587877; doi:10.1016/j.dib.2017.08.033)

*
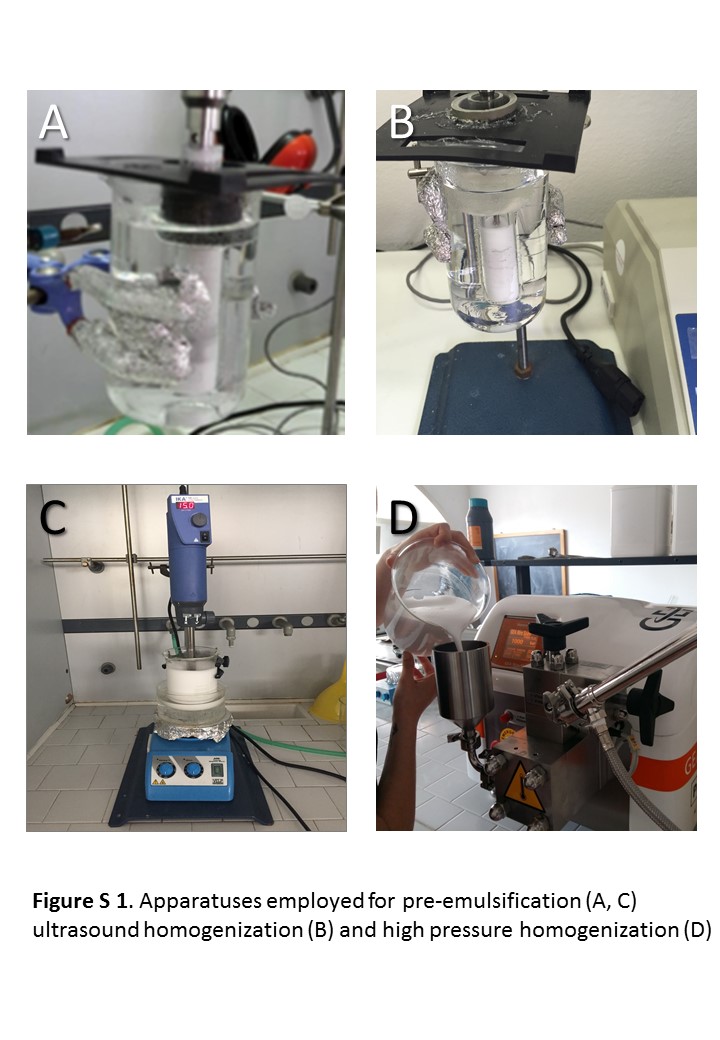

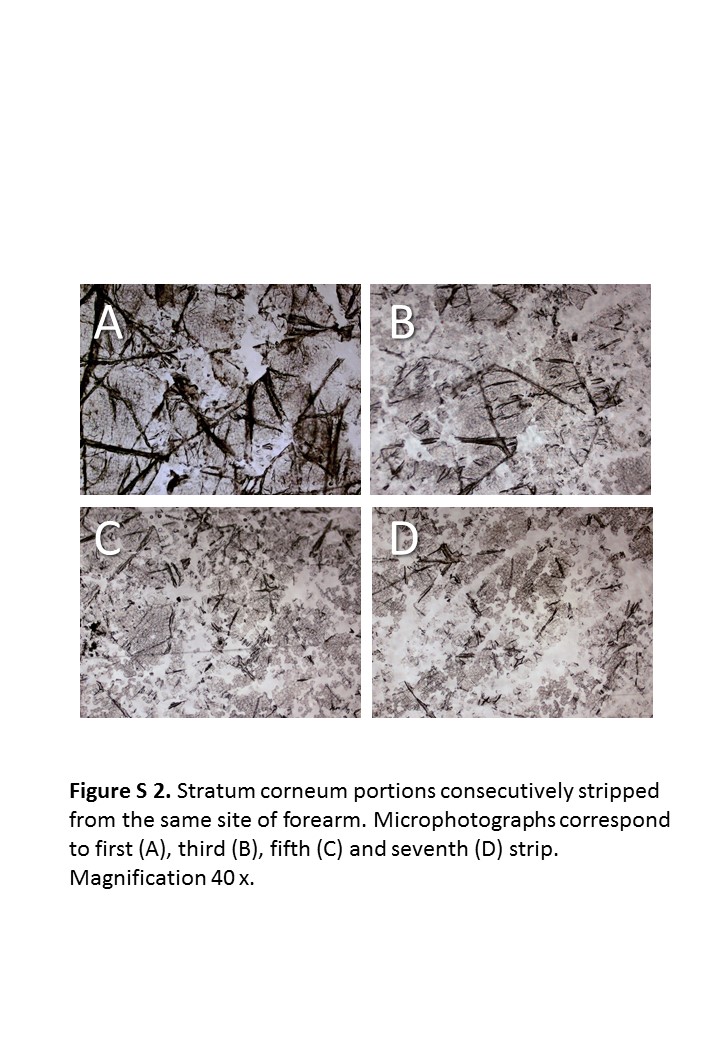

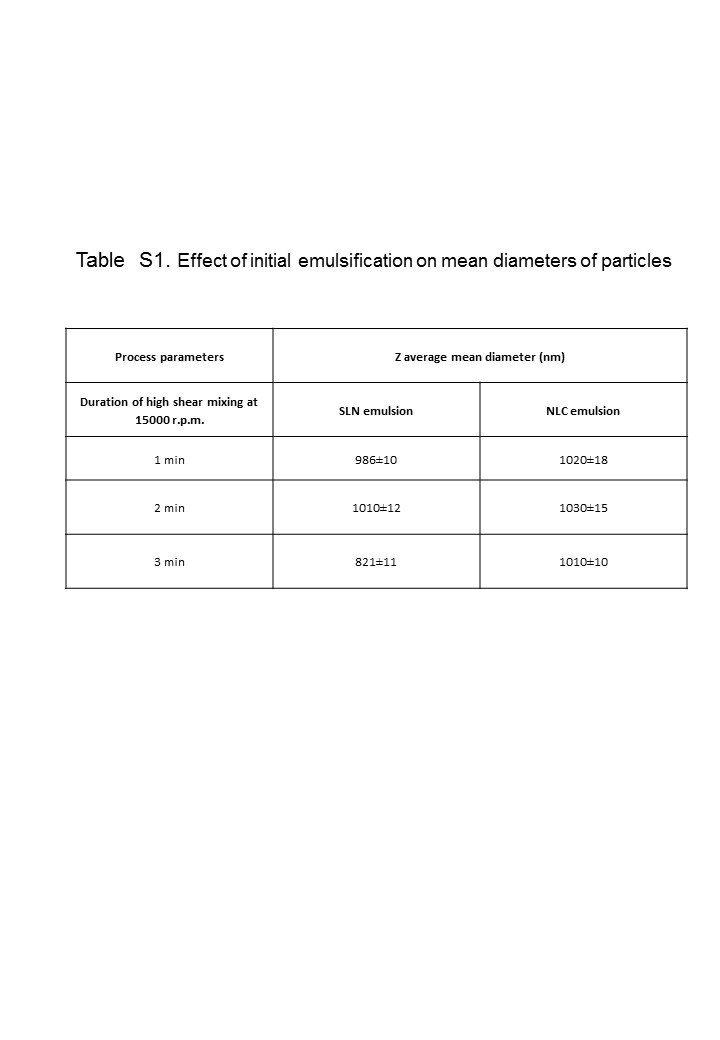

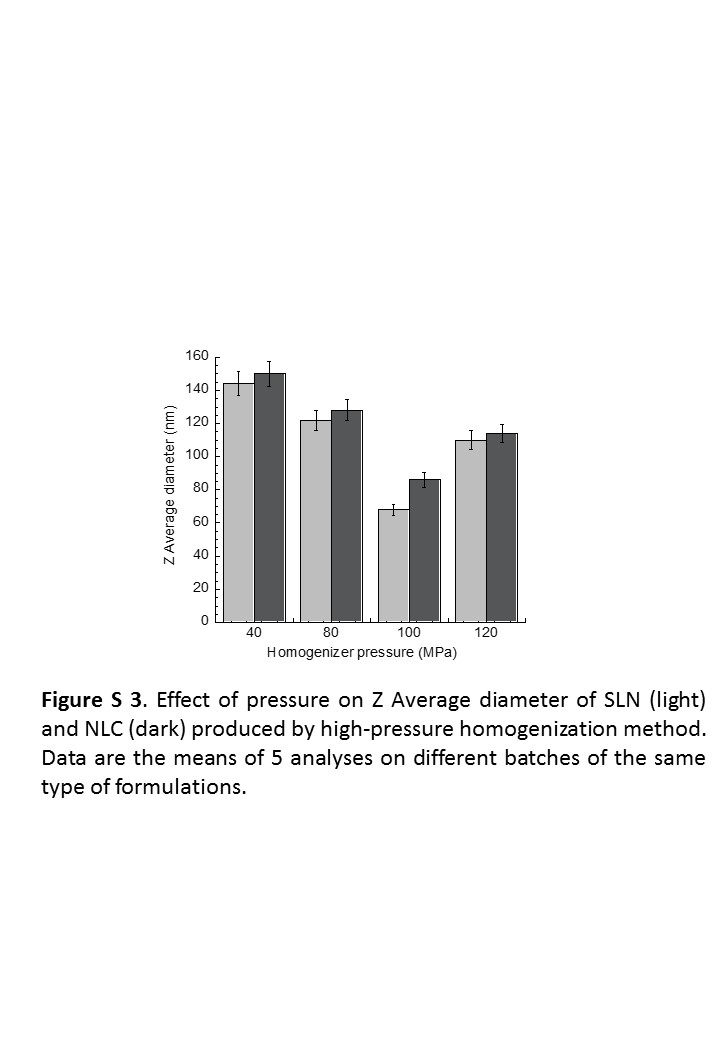

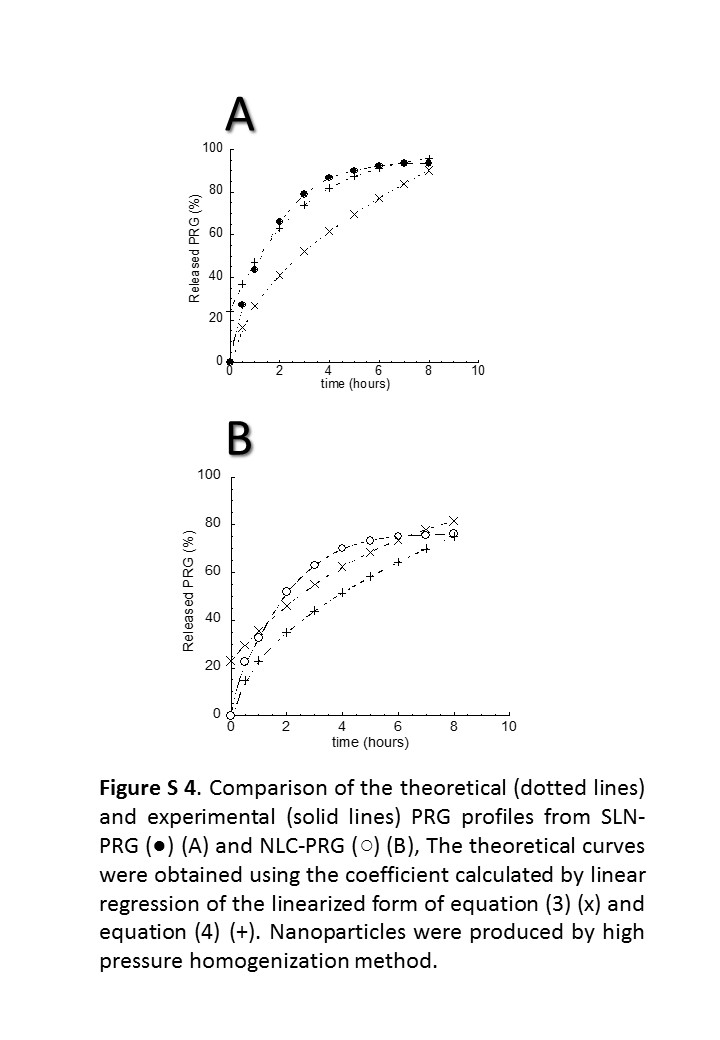
*

Supplement: Supplementary file 1 — Supplementary material [file mmc1.docx]
